# Emergency Department Virtual Happy Hour: A Novel Approach to Peer Group Support During a Pandemic

**DOI:** 10.7759/cureus.35321

**Published:** 2023-02-22

**Authors:** Adeola A Kosoko, Shabana Walia, Ryan Huebinger

**Affiliations:** 1 Department of Emergency Medicine, UTHealth Houston, Houston, USA

**Keywords:** postgrad medical education, covid-19 pandemic, wellness resources, wellness and resilience, social support

## Abstract

Introduction

Communities responded to the coronavirus disease 2019 (COVID-19) pandemic with mandatory social-distancing regulations. Pandemic and disaster research shows that social isolation can often cause negative emotions and medical provider burnout. The primary objective of this study was to create and evaluate a novel wellness program, the Happy Hour Wellness Initiative (HHWI), based on peer support group concepts to foster resilience for emergency healthcare providers in response to a novel disaster.

Methods

The study was performed at a large emergency department with physicians, advanced practice providers, and staff invited to attend weekly “virtual happy hour” sessions. Participants individually opted into each of the six weekly sessions, with no obligation to attend. The program was designed based on the tenets of a peer support group and implemented by video conferencing. Participants completed a demographic questionnaire and answered open-ended questions after the six-session HHWI ended.

Results

Of the 40 survey respondents, 30% reported feeling stressed and 40% felt isolated at the early declaration of the pandemic. Regarding the HHWI, 90% of participants had no expectations from the HHWI, but 90% reported that their favorite part of the initiative was the feeling of togetherness. Most participants (95%) requested a continuation of the HHWI, even if not by a video-conferencing platform, and 90% reported feeling sad after the HHWI ended.

Conclusion

The emergency department HHWI was a welcome opportunity for employees to combat stress and anxiety brought on by the COVID-19 pandemic and social distancing. The initiative fostered team building, comradery, group advocacy, stress relief, and cheerfulness. The initiative was so greatly welcomed as a tool for wellness that almost all participants recommended that the HHWI should be available, not just in times of hardship, but year-round. The HHWI has provided a new approach to promote wellness in emergency care providers using a peer support group.

## Introduction

A disaster is an unfortunate natural or human-induced hazard that negatively affects a society or the environment. Some disasters are predictable, allowing enough time to either prepare for the event or flee; some are brief or barely noticeable on a broad scale; and yet others are unpredictable and thrust people into unforeseen circumstances. Nonetheless, the greater the scope of impact or personal loss, the greater the psychosocial effects [1]. During the initial phases of a disaster, people often reckon with feelings of confusion, grief, and sometimes anger. An individual’s coping mechanisms have been cited as an important factor in helping disaster victims adjust to a dramatically new environment. Coping is defined as “constantly changing cognitive and behavioral efforts to manage specific external and internal demands that are appraised as taxing or exceeding the resources of the person [2].”

On March 11, 2020, the World Health Organization declared coronavirus-19 (COVID-19) a pandemic. On March 13, 2020, the United States declared a national emergency in response to the COVID-19 pandemic. Concerns about the virus spreading led to many communities instituting shelter-in-place orders. Various local and state governments mandated what was a new concept to many people - social distancing, meaning limiting physical contact/proximity of persons to slow the spread of a highly contagious disease. In addition, people who did not need hospitalization but were potentially exposed to the virus were ordered to maintain medical quarantine at home. Although a limited number of studies have investigated pandemics to date, their findings have consistently shown that isolation can lead to anxiety, depression, and anger [3-5]. Moreover, this current pandemic is a health “disaster of uncertainty.” Day by day, experts are describing new findings and making new recommendations, especially in the early stages of its recognition. Disasters of uncertainty have the potential to generate the greatest number of psychological casualties [6].

Mental health professionals are often concomitantly deployed with disaster relief efforts because of the potential for significant psychological impact. Mental health interventions help individuals build resilience, take steps to meet goals, and connect with other people to develop coping mechanisms [7]. However, the availability of qualified mental health providers can be sparse during a pandemic, particularly when social distancing is necessary. Other techniques may also be used to promote mental health, including support groups, which have been beneficial for people experiencing crises of addiction or chronic disease. Compared with other physicians, emergency physicians are at a baseline higher risk for burnout because of the system, culture, and society in which they practice [8]. Emergency medicine (EM) has received attention in both academic circles and popular medical literature for the high rates of burnout among EM physicians and trainees [9]. Building perseverance in EM is viewed as such an essential quality that the Accreditation Council for Graduate Medical Education formally recognized the importance of well‐being in trainees, with many EM programs seeking curriculum adaptations to combat burnout [10].

Emergency physicians and other frontline healthcare providers are particularly vulnerable to the negative mental health effects of the COVID-19 pandemic and social distancing. These individuals balance the duties of caring for others in their most critical time of need but also can often experience distressing concerns for their own well-being and that of their loved ones, with new restrictions on their typical coping mechanisms. An unfortunate example in the EM community was the loss of Dr. Lorna Breen, an emergency physician, by suicide, though motivations were multifactorial, which occurred early in course of the pandemic [11]. Although studies have shown that wellness curricula can be useful to foster wellness during residency training [12-14] little information is available about wellness initiatives for healthcare workers during a disaster, particularly during a pandemic.

Peer support groups occur when people with something in common utilize each other for help, connection, or reciprocity. There is no one formula for forming a peer support group, but there are expectant themes: composed of peers, have a facilitator, and give equal opportunity to participate. They have been noted to be beneficial in an assortment of situations to promote coping and overcoming conditions and experiences (e.g., addiction, bereavement, disease, parenting, identity, and others) [15].

Understanding the innate higher risk for poorer psychological outcomes for frontline healthcare providers during the COVID-19 disaster, we wanted to combat burnout and promote wellness for the employees in our emergency department. We designed a novel program, the Happy Hour Wellness Initiative (HHWI), based on the concepts of a peer support group. The primary aim of our project was to create and present a novel wellness initiative for emergency healthcare providers to foster coping in response to the COVID-19 pandemic and then to evaluate the effects of this program.

## Materials and methods

Study setting and participants

This study was a single-center retrospective study conducted in a large urban academic center that hosts a three-year EM residency. A total of 115 attending physicians and those in fellowship training, 60 resident physicians, 55 advanced practice providers, including nurse practitioners and physician’s assistants, and 32 office staff were all members of the department and invited to participate in the HHWI. Invitations were distributed by a department-wide email with a flyer and an electronic calendar invitation. The employees received the invitation five days before each event and again on the day of the HHWI session, with an access link to the video conference if they chose to participate. Of those invited, there was a range of 12-33 participants logged onto the video conference at any given time. Everyone in the department was also invited to complete the survey at the completion of the HHWI.

Intervention

Each week, the participants received invitations to participate in a one-hour HHWI session. Each session was held using Cisco Webex Meetings (version 39.8, Milpitas, CA) video-conferencing platform. We designed our own initiative de novo, as there was limited research on the subject matter. There had been limited validated research pertaining to healthcare worker wellness initiatives, particularly during times of disaster. A total of six HHWI sessions were offered over six weeks during the initial shelter-in-place orders for the state. We produced one session per week during the first six weeks of the stay-at-home order in Harris County (March 24, 2020-May 1, 2020). On May 4, the stay-at-home order was amended as Harris County implemented a safe phased reopening of services. We ceased the wellness initiative to assess the value of the program considering people now had more options and coping mechanisms than at the commencement of the HHWI. For each of the sessions, the participants self-selected and participated to the level of their comfort, availability, and/or interest (e.g., video versus audio, chat messages versus oral discussion, and active participation versus observation).

Each session was structured similarly with two segments “hosted” by the researchers, who are each emergency physicians and do not have specialized mental health or wellness training. Each session began with a 45-minute segment focused on the state of individual and group wellness, sharing wellness tips and looking for wellness solutions (called “Well, Well, Well”). This first segment was followed by a 15-minute segment meant for fun and entertainment (called “The Quarantini Quarter Hour”). Any presenters during the Quarantini Quarter Hour were volunteers from the emergency department who prepared their contribution before the HHWI session. Participants could join or leave the HHWI freely at any point during a session or during the six-week course. Similarly, participants could join the HHWI at any time. There was no obligation to participate.

The entire department, regardless of participation or engagement in the HHWI, was equipped with free-of-charge formal mental health (i.e., counseling, psychiatry) service options should they want to utilize such offerings at any time.

Outcomes

The outcomes studied were the results of the field notes kept by the researchers from wellness sessions and the participants’ open-ended responses to the six non-demographic survey questions.

Data collection and analysis

During the wellness sessions, field notes and observations were recorded by the researchers/wellness hosts with the intention of distributing information regarding resources to those who could not attend and to improve on future sessions. The notes were bulleted observations and shorthand. The notes were compiled into a deidentified summary for each session. The sessions themselves were not recorded in order to protect participants’ privacy and encourage a safe, open environment for discussion and sharing. In addition, five days after the completion of the sixth, and final, session of the HHWI, a nine-question survey was sent to the entire EM department with a consent letter and an optional invitation to participate in the research questionnaire. There were two email reminders sent to encourage survey participation, each one week apart. The surveys were conducted using a secure electronic survey software system (Qualtrics, Provo, UT). The initial three questions collected demographic information, including age, sex, and the participant’s role in the department. The remaining six survey questions were related to the participant’s attitude toward the HHWI and the social-distancing mandate: two topics that the investigators felt would be important to guide future wellness initiatives. All the survey responses were uploaded to NVivo software (version 11, Melbourne, Australia) verbatim for analysis. The main themes and topics were identified using word frequencies and coding schemas. We identified the top three themes for each inquiry in the questionnaire and calculated the percentage of representation compared to the total responses [16]. The deidentified field notes were collected and summarized by the lead investigator (AK) and any common themes were noted.

This study was approved by the University Institutional Review Board. Though the field notes were initially taken for quality improvement in the wellness initiative, the IRB also approved them for inclusion in study analysis in a request for appraisal of the overall HHWI.

## Results

Demographic data

**Figure 1:**
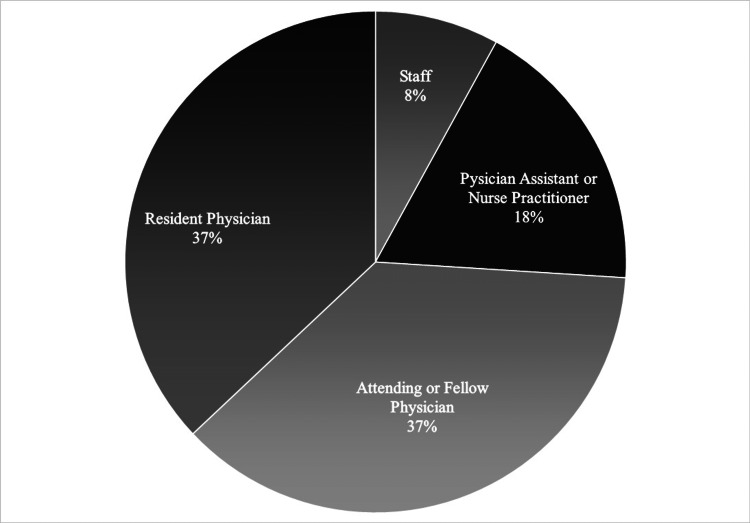
Participants’ Roles in The Emergency Department (N=78)

Seventy-eight employees participated by attendance in the HHWI in some capacity, with a range of 12-33 people present in each session. The survey generated 40 responses from different employee groups as follows: 15 resident physicians; 15 attending or fellow physicians; seven advanced practice providers; and three office staff (Figure 1). Most respondents were women (65%) (Table 1).

**Table 1:** Demographic Characteristics of the Questionnaire Participants (N=78)

Demographic characteristics		Proportion
Sex of respondents	Male	35%
	Female	65%
Age of respondents	25-34	37%
	35-44	42%
	45-54	15%
	55-64	6%

Results of researcher field notes

Field notes were kept by researchers during the HHWI, including the direct quotes of participants (Table 2).

**Table 2:** Field Notes of Observations During the Happy Hour Wellness Initiative Sessions APP, Advanced Practice Provider; CDC, Centers for Disease Control; COVID-19, Coronavirus 2019

	“Well, Well, Well”	“Quarantini Quarter Hour”	Observations
Week 1	Open forum	None	“Honestly…I’m scared.” Review of newest CDC recommendations Reassurance of available personal protective equipment from administration Hospital-laundered scrubs became ubiquitously available for all healthcare providers as a result of discussion 3 people shared how they keep from spreading hospital exposure to their families
Week 2	How are you coping? What are your tips and tricks? How are you staying fit? What restaurants would you recommend? Does anyone have any extra resources? Is anyone in need of any resources?	Self-Defense moves, music talents (guitar, saxophone, singing), growing a vegetable garden, skit	An offshoot exercise challenge within the department developed “I know where to get toilet paper in the city” One person was livestreaming the session during a clinical shift. “Thank you for involving the APPs”
Week 3	How are your fitness goals progressing? What are some good things that you hope persist when this is over? What are you drinking? Happy Birthday wishes for the month. Introduction of mental health resources available through the University, national organizations, and privately	“Name that Provider” game Yoga lesson	One person was actively exercising on a Peloton bicycle Group Exercise in gratitude: ● More time with family ● Less traffic ● Renewed interest in medicine ● Food donations to the workers About 50% of participants brought a beverage without prompting, about 25% of participants had an alcoholic beverage Developed a resource webpage for the department to consolidate information One person was livestreaming during a clinical shift 3 providers actively making “ear savers” for employees to ease wearing masks Drinks included water, tea, soft drinks, milkshake, juice, alcoholic beverages
Week 4	What are some restaurant suggestions? How are your fitness goals coming along? What are some podcasts, movies, or TV shows that you would recommend?	Show off your collection (hot sauce collection, whiskey collection)	“When this is done, we should have a journal club at your house.” Several people seemed to be livestreaming during a clinical shift “I have an idea for a session!”
Week 5	Let’s make a music playlist based on topics associated with COVID that we can share as a department.	Mixology lesson, show off pet tricks High School students read letters of support to the group.	The entire department, even nurses and technicians and providers who could not attend the sessions contributed to the music playlist. The result was a 120-song playlist of multiple genres that was shared among the group.
Week 6	Alumni Day. Tell us where you came from and how you became an “essential worker”	magic trick, painting lesson, Arabic lesson	Each person who shared their story had careers trajectories that would not intrinsically suggest a career in medicine (i.e. athlete, magician, artist, and writer). “Are we meeting next week?”

Survey responses

Three main themes emerged in the analysis of the participant’s feelings about the social distancing mandate (Table 3). The most frequent descriptors that participants used in answering the survey question were “stressed,” “isolated,” and “happy.” The participants stated that their stress was most often related to anxiety and fear for their family and friends because of the potential risk of infecting them with the disease. Others felt stressed and frustrated with the government's responses to the pandemic. Some mentioned that their stress was caused by changes in their lifestyle associated with social distancing, such as not being able to visit friends at their houses or being able to go shopping. “Isolation” was another word that was mentioned very often in participants’ responses to this question. However, for some participants, the distancing mandate was not altering their everyday routines. In addition, other participants felt able to focus more on their work than before the pandemic, which was making them feel “happy.”

**Table 3:** Major Themes and Exemplary Quotes from the Happy Hour Wellness Initiative Survey CDC, Centers for Disease Control; COVID-19, Coronavirus 2019; EM, Emergency Medicine

Question	Major Identified Themes	Notable Quotes
Please describe how you felt at the onset of the rapid spread of COVID-19 with government mandates for social distancing.	Stressed 80%, Isolated 40%, Happy 20%	“I was mostly concerned how we’d keep ourselves safe”, “Honestly very disappointed”, “Stressed because of uncertainty about what to do/not do. Recommendations were changing so frequently”, “I was worried about my family, friends, and myself”, “unsure, lonely, not sure what to do with my extra time”
What (if any) were your expectations for the EM Happy Hour Wellness Initiative?	No Expectations 90%, Stress Relief 81%, Connection 76%	“Share comfort and concerns with people who understand and won’t be alarmed at what we tell them”, “A coming together during a stressful time”, “Way to remain connected with colleagues”, “Forgetting all the craziness going on”, “Opportunity to laugh and see people we otherwise couldn’t see”, “Social interaction and decompression”
What was/were (if any) your favorite aspect(s) of the EM Happy Hour Wellness Initiative?	Being Together 90%, Music/Showcase 45%, Nothing Particular 20%	“Provided time just to be silly and decompress”, “I liked brainstorming the music list”, “Seeing attendings have fun”, “Seeing people’s talents or collections, getting to know them better”, “The group openly discussing their worries”, “I liked that people had an opportunity to talk about how they felt. I also liked that there were scheduled events to entertain us.”
How would you improve the EM Happy Hour Wellness Initiative?	Continue 95%, Timing 40%, Additional Activities 20%	“I hope this is continued in some way in the future. Could it be used for faculty development?”, “I want to learn more about people in the department”, “Extend to in-person happy hour maybe once a month”, “More inclusion of the group”, “Continue having directed activities”
Please describe how you felt at the lifting of the governmental social-distancing mandate.	Worried/Concerned 90%, Happy/Glad 85%	“Extremely disappointed”, “Too early with no guidelines on how to safely proceed”, “Positive – allowed me to get some of my routine back”, “Liberated. But still dangerous”, “Concerned about the spread of infection”, “I feel the same. I’m not going anywhere yet. I don’t feel safe yet.”
Please describe how you felt about the cessation of the EM Happy Hour Wellness Initiative.	Sad 90%, Served its Purpose 75%, Not Attended 15%	“Sad! It’s a great source of support that we won’t have anymore”, “Consider restarting as an option to stay connected”, “Sad, I wish it kept going.”, “I miss it a little”, “Disappointed”, “Happy it could happen in person!”

The next survey question related to the participants’ expectations of the HHWI prior to ever attending a session (Table 3). Analysis showed that most participants (90%) had no specific expectations of the initiative. Some participants (81%) mentioned that they hoped the HHWI would allow them to relieve the stresses they were experiencing, particularly associated with COVID-19 and social distancing. Another dominant theme was participants (76%) explaining that they were looking forward to socializing, an activity that was directly being challenged by the social distancing mandate, with colleagues during the HHWI.

When asked about their favorite aspect of the HHWI (Table 3), the factors mentioned most often were “being together” (90%) and “music/showcase” (45%); also, a few participants (20%) cited “nothing in particular.” Many respondents stated that they enjoyed being social with colleagues thanks to the HHWI. Specifically, they enjoyed seeing colleagues without face-covering masks, having conversations that were not related to COVID-19, and being more “normal.” Many participants stated that this initiative allowed them to learn more about their colleagues as fellow human beings through basic conversation.

When participants were asked questions about potential improvement for the HHWI (Table 3), the improvement most often mentioned was to simply continue the initiative (95%). Many participants felt that the initiative should continue regardless of the pandemic or social distancing: “I hope this is continued in some way.” Some suggested decreasing the frequency to once monthly, whereas others felt that the weekly activity was preferable. Other participants suggested that the emergency department continue the same activities in person once the social-distancing mandate is lifted. Others felt that it would be good to continue the HHWI using teleconferences. Some suggestions were that integrating an exercise competition or playing games would improve the initiative. Interestingly, two participants suggested integrating more alcohol use in the form of playing “drinking games” or having alcoholic beverages delivered to them for the event. Regardless of the proposed ideas, almost all the survey participants (95%) said that the initiative should continue because it helped participants relieve stress, anxiety, and frustration while teaching them more about their colleagues and helping them to overall feel united. The latter aspect of togetherness was emphasized in many responses, which suggested that feeling connected during the time of social distancing was an important coping mechanism: “It gave us something to be happy about.”

The responses to the question “Please describe how you felt at the lifting of the governmental social distancing mandate” showed that the EM employees were simultaneously happy (85%) and worried (90%) (Table 3). Almost all respondents mentioned that they were happy about the fact that these restrictions were lifted; however, almost all of them mentioned that they were worried about the potential consequences of the decision. For example, one of the participants stated, “glad to get the chance to move a little freer, but also concerned about things getting worse.” Another participant said, “happy, but nervous we [could] have another surge.”

The final question of the survey was about participants’ feelings when the HHWI ended (Table 3). The most common theme to emerge in the analysis was a feeling of sadness (90%) that the initiative was over. But there was a hope to participate again in an HHWI in the future: “It was a great source of support that we don’t have anymore.” Many respondents were grateful to the organizers for their effort in establishing the sessions and presumed that the initiative served its purpose. “It was fun while it lasted” and “…all good things must come to an end.” Nonetheless, others were optimistic about the end of the virtual meetings and encouraged that they would evolve as one participant stated, “happy it can now happen in person!”

## Discussion

In response to a national and global disaster, the COVID-19 pandemic, we created a wellness initiative structured for emergency healthcare providers. We wanted to create an intervention for the anticipated feelings of distress and to address the contributors to burnout that would likely disproportionately affect an emergency care provider in a time of acclimation to a new lifestyle and the work regimens created by a disaster.

Studies have shown that pandemics can cause stress to those affected by a disaster [17]. Unsurprisingly, social distancing was a major cause of social isolation within our EM group. Therefore, we predicted, that our department would benefit from a group-centered intervention - a group setting to pursue wellness. We facilitated a healthy discussion regarding the negative effects that the pandemic was having on personal and group wellness, discussed problem-solving, shared resources, and socialized with the goal of building resiliency. Specifically, the HHWI was successful in providing a platform to discuss feelings, share coping strategies, motivate one another to continue working, empower participants to keep themselves and their loved ones safe, provide hope, clarify understanding of the situation, and provide feedback to the administration about needs and concerns in the department. In summary, although the HHWI was advertised as a place for a group of emergency providers to pursue wellness, it ultimately functioned as a peer support group.

We did not anticipate that the HHWI would be so well received. We were surprised by the predominant theme of participants’ feedback to continue the intervention. Regardless of the pandemic or social distancing mandates, discontinuing the program elicited sadness from participants because they enjoyed the program so much. This finding suggested that not only are peer support wellness initiatives important for emergency practitioners to prevent burnout [18] but interventions such as this program are also desired by practitioners, regardless of outside stressors such as a pandemic.

A few reports have documented physicians or other healthcare providers using support groups to address general provider wellness [19]. One study used a peer support group format for residents based on discussions over ice cream [20]. Since the onset of the pandemic, support groups have been successful in promoting wellness for non-providers during the pandemic [21]. There are studies evaluating the impact of the pandemic response on wellness [22], but despite the negative impact of the pandemic on physician wellness, studies on wellness support initiatives during a pandemic are limited. One study found that a single support group session was very well received in supporting physicians through a pandemic. While a subset of participants felt that their mental wellness was negatively impacted by the session, all participants felt that the sessions improved their social wellness [23]. The positive feedback that we received agrees with these findings that a support group format is appreciated by emergency department workers as a method of promoting coping during a pandemic and social distancing. Future research could point to other more structured wellness initiatives.

The circumstances of the pandemic created several unique aspects for our initiative. Specifically, participants of any support group should have shared characteristics. The unifying characteristics of our group were members of the same academic medical department and a group of individuals experiencing life responding to a global pandemic. Nonetheless, participants’ roles in the department varied greatly. We also uniquely used telecommunication, rather than in-person meetings, which was very useful considering the government mandate that required social distancing. Though the interactions did not utilize physical proximity, simply having the opportunity to see people and participate in friendly interactions was quite beneficial to many participants. Interestingly, the HHWI also did not use a professional mental health facilitator. Support groups do not require a professional facilitator to be beneficial. Our group was self-reliant and the peers met the needs of others in the group. The facilitators operated as peers who simply kept the conversation flowing and on track. The only notes that participants made regarding the facilitators was to thank them; there was no mention of requesting a professional. Nonetheless, personal complimentary professional mental health services were made available to any desiring member of the department separate from the HHWI sessions.

Interestingly, 15% of survey participants who filled out the survey also reported that they did not attend the HHWI sessions. We do know that the HHWI gained popularity in the ED such that it would often be playing in work areas in the ED so that all physicians and advanced practice providers (APPs) working were able to overhear the sessions. Everyone in the department also had access to the music playlist developed from the HHWI. Another consideration is that with a large reduction in social outlets at the time, the HHWI became something that was often discussed outside of the HHWI, and the 15% of people may have been filling out the surveys based on their adjacent experiences or based on what they were seeing occurring with colleagues despite not personally signing on to participate in the HHWI. Unfortunately, there was no mechanism to better understand this outcome.

Of note, although the theme of the initiative was virtual “Happy Hour,” which is a concept very commonly associated with alcohol, drinking alcohol was not a dominant theme of our initiative based on researcher observation and participant feedback. Nonetheless, a minority of participants consumed an alcoholic beverage during the session and a few of the session activities included some discussions about alcohol. Only two participants gave feedback requesting that alcohol play a greater role in the HHWI. It is important that alcohol is not a driving influence of a wellness activity such as the HHWI. Emergency physicians account for 7-18% of physicians enrolled in programs for substance abuse management [24]. The “happy” in the HHWI indicates wellness, resilience, and health; it does not advocate for alcohol as a coping mechanism. The structure of the group could potentially be advantageous for the identification of and intervention for substance abuse disorders in healthcare providers if it is to be examined further in the future.

Although the HHWI ultimately played the role of a support group, for some participants it seemed to also provide an avenue for entertainment. For some participants, it served as a place for advocacy for the healthcare providers during the many uncertainties within the medical system during a pandemic. Another effect of the HHWI was team building, as many participants described “getting to know” each other better.

Limitations

This study is limited by the design to evaluate a single, university-based, emergency department with self-selected participants; a multi-centered analysis would provide a more robust evaluation. We measured our outcomes with qualitative data, which were analyzed by a single researcher. Quantitative comparison with a control group could provide additional, useful insights. Additionally, we did not quantitatively evaluate well-being before or after the sessions and did not have another formal evaluation of the sessions, so we are unable to objectively quantify the efficacy of the initiative. A major limitation of the data collection and analysis was the lack of recorded and transcribed sessions and a lack of a second coder or adjudicator reviewing the quantitative data.

The structure of the sessions was also casual, aside from the two designated segments composing the HHWI; this could limit the ability of other institutions to precisely replicate the process and results. However, the casual nature of the sessions made the setup and maintenance very approachable and customizable to various groups of people. Future research should explore whether peer support groups are a practical alternative to foster emergency provider wellness year-round and compared to times of crisis. It may also be beneficial to evaluate whether this type of wellness initiative may be beneficial in other academic medicine or surgical departments. Participation in the sessions may have been influenced by the nature of the pandemic, as outside activities were limited during the lockdown. While this may have increased the participation in such an initiative in comparison to a time when such stressors are not present, other initiatives have found similar success outside of a pandemic. In comparison to prior wellness initiatives, the virtual nature of the presented initiative may have benefits in improving flexibility and participation by participants. With limited research on peer-support groups and the impact of COVID-19 on provider wellness at the time, many of the survey questions were intended to evaluate specific concerns relating to the pandemic and to guide future wellness initiatives. From our results, future evaluations of wellness initiatives can generate more thoughtful and directed questions. Only 52% of participants completed the survey. This could introduce bias in our survey results, as participants that were more enthusiastic about the wellness initiative may have been more likely to complete the survey. Additionally, participants with higher levels of burnout may have been less likely to complete the survey. The evaluation of the field notes was performed by one investigator, and a more thorough evaluation by multiple investigators could have potentially strengthened the findings.

We included non-physicians in our initiative, which could be viewed as a barrier to achieving physician peer support. Interestingly, while clinical stressors were certainly a topic of discussion during these sessions, many discussions centered around non-clinical items, and human experiences, which were ubiquitously experienced by physicians and non-physicians alike. Hence, we feel that the inclusion of non-clinicians not only allowed more individuals to access this potentially beneficial initiative but also enriched the discussion.

## Conclusions

This study confirmed that the pandemic and resultant social-distancing mandates had a negative impact on the emergency department members in this study. Those who participated in the HHWI were grateful for the opportunity to experience fellowship and feel the support of their colleagues in a time of stress and uncertainty. Many described how they found joy in participating in the initiative. The HHWI was so that it may provide a new framework, not just for facilitating wellness and coping during a pandemic, but also for bolstering wellness in general for emergency care providers.
